# The Role of 18F-FDG PET/CT in Monitoring Immunotherapy Response in Non-Small Cell Lung Cancer: Current Evidence and Challenges: A Narrative Review

**DOI:** 10.3390/diagnostics15212754

**Published:** 2025-10-30

**Authors:** Roxana Mladin, Cristian Oancea, Emil Robert Stoicescu, Agneta Maria Pusztai, Amalia Constantinescu, Emanuel Poplicean, Diana Manolescu

**Affiliations:** 1 Doctoral School of ‘Victor Babes’ University of Medicine and Pharmacy Timisoara, Eftimie Murgu Square 6 No. 2, 300041 Timisoara, Romania; roxana.mladin@umft.ro (R.M.); amalia.constantinescu@umft.ro (A.C.); emanuel.poplicean@umft.ro (E.P.); 2 Center for Research and Innovation in Precision Medicine of Respiratory Diseases (CRIPMRD), ‘Victor Babes’ University of Medicine and Pharmacy Timisoara, 300041 Timisoara, Romania; oancea@umft.ro (C.O.); dmanolescu@umft.ro (D.M.); 3 Department of Pulmonology, ‘Victor Babes’ University of Medicine and Pharmacy Timisoara, 300041 Timisoara, Romania; 4 Radiology and Medical Imaging University Clinic, ‘Victor Babes’ University of Medicine and Pharmacy Timisoara, Eftimie Murgu Square No. 2, 300041 Timisoara, Romania; 5 Research Center for Medical Communication, ‘Victor Babes’ University of Medicine and Pharmacy Timisoara, Eftimie Murgu Square No. 2, 300041 Timisoara, Romania; 6 Research Center for Pharmaco-Toxicological Evaluations, ‘Victor Babes’ University of Medicine and Pharmacy Timisoara, Eftimie Murgu Square No. 2, 300041 Timisoara, Romania; 7 Field of Applied Engineering Sciences, Specialization Statistical Methods and Techniques in Health and Clinical Research, Faculty of Mechanics, ‘Politehnica’ University Timisoara, Mihai Viteazul Boulevard No. 1, 300222 Timisoara, Romania; 8 Department of Anatomy and Embryology, ‘Victor Babes’ University of Medicine and Pharmacy Timisoara, Eftimie Murgu Square No. 2, 300041 Timisoara, Romania

**Keywords:** 18F-FDG PET/CT, immunotherapy response assessment, non-small cell lung cancer, iRECIST and PERCIST criteria, metabolic tumor biomarkers

## Abstract

**Background/Objectives:** Non-small cell lung cancer (NSCLC) remains a leading cause of cancer-related mortality worldwide. Immune checkpoint inhibitors (ICIs) have transformed treatment paradigms, but assessing response remains challenging due to atypical patterns such as pseudoprogression, hyperprogression and dissociated response. Conventional evaluation criteria, such as RECIST 1.1, may not fully capture these patterns, leading to potential misclassification and premature therapy discontinuation. This review explores the role of 18F-FDG PET/CT in assessing immunotherapy response and highlights novel imaging criteria to enhance clinical decision-making. **Methods:** A systematic literature review was conducted across PubMed, Web of Science, Scopus, and Cochrane Library, selecting relevant studies published between 2013 and 2024. The review focuses on the strengths and limitations of PET-based imaging in monitoring NSCLC immunotherapy outcomes. Specific attention was given to evolving evaluation frameworks, including iRECIST, PERCIST, imPERCIST, and iPERCIST, as well as metabolic biomarkers such as metabolic tumor volume (MTV) and total lesion glycolysis (TLG). **Results:** Compared with anatomical-based assessment, metabolic imaging using 18F-FDG PET/CT may offer deeper insights into tumor behavior during immunotherapy. PET-derived parameters seem to improve the detection of immune-related response patterns, providing a more refined approach to differentiate true progression from pseudoprogression. Emerging evidence indicates that metabolic biomarkers such as metabolic tumor volume (MTV) and total lesion glycolysis (TLG) could serve as useful predictors of therapeutic efficacy and support treatment adaptation. Nevertheless, current findings are mainly based on small, heterogeneous, and predominantly retrospective studies, with variable PET timing and threshold definitions that limit the generalizability of these results. **Conclusions:** 18F-FDG PET/CT represents a promising complementary tool for assessing immunotherapy response in NSCLC. Its integration with advanced imaging criteria and metabolic tumor biomarkers may enhance response evaluation and assist clinical decision-making. Nonetheless, the current evidence remains preliminary, and further standardization and large prospective validation studies are required before its routine implementation in clinical practice.

## 1. Introduction

Globally, lung cancer is the most commonly diagnosed malignancy and the leading cause of cancer-related mortality in both sexes [1,2,3], with a 5-year survival rate ranging between 10 and 20% [1,4]. The prognosis of lung cancer is influenced by factors such as the histopathological subtype, tumor characteristics, and disease stage [1,5].

The latest rapid advances in oncology have made the dream of precision medicine into a reality, with targeted therapy available for various cancers depending on the molecular genotype. This progress has also led to the development of personalized radiology, involving distinct tumor response criteria designed to aid in the assessment of disease response or progression based on the therapy employed [6,7].

In recent years, the success of systemic immunotherapy for cancer treatment has transformed the field of oncology, shifting perceptions toward a more optimistic outlook and significant interest within the medical community. Mechanistically, all aspects of immunotherapy share a common focus on the key mechanisms of the tumor microenvironment (TME). A particular importance focuses on overexpression of immunosuppressive checkpoints, such as cytotoxic T-lymphocyte-associated protein 4 (CTLA-4), Programmed Death 1 (PD-1), and Programmed Death-Ligand 1 (PD-L1) within the TME, which can impair the immune system’s ability to destroy cancer cells [8,9]. Various systemic immunotherapies against cancer have been developed, among which, the most interesting are inhibitors of the immune checkpoints—ICI (monoclonal antibodies directed against immune checkpoints) [9,10]. While ICIs have demonstrated improved overall survival and better disease control across numerous oncological pathologies, they are effective for only 38.5% of patients with metastatic solid tumors or hematologic malignancies [8,11]. Nevertheless, these therapies remain limited by the low proportion of patients achieving an objective tumor response, the occurrence of systemic adverse effects related to immunotherapy, and the emergence of long-term resistance [12,13,14,15].

Due to the unique mechanisms of action based on T-lymphocyte activity, post-immunotherapy responses are characterized by distinct patterns, including pseudoprogression, hyperprogression, and dissociated response [8,9]. Although the clinical benefits and evaluation of immunotherapy responses remain ambiguous, the immune Response Evaluation Criteria in Solid Tumors (irRECIST) were proposed as an update to the RECIST 1.1 criteria to better assess post-immunotherapy responses [16].

So far, five sets of tumor response evaluation criteria have been developed to more accurately assess changes in the size of target lesions, incorporating the specific response patterns associated with immunotherapy. These include the immune-related response criteria (irRC), immune-related RECIST (irRECIST), immune RECIST (iRECIST), immune-modified RECIST (imRECIST), and intra-tumoral RECIST (itRECIST) [8].

Recently, significant progress has been made in the diagnostic and treatment options for patients with lung cancer, particularly non-small cell lung cancer (NSCLC). These advances include: 2-deoxy-2-[18F] fluoro-D-glucose positron emission tomography/computed tomography (FDG PET/CT) for treatment staging and response assessment [17], targeted radiotherapy, molecular immunotherapy [18], minimally invasive endoscopic biopsy, and minimally invasive surgery [19].

Currently, FDG PET/CT is part of the routine staging process for NSCLC. Moreover, there is growing interest in utilizing FDG PET/CT metrics to estimate the response to ICI therapy. While standard uptake values (SUVs) used in FDG PET/CT have limitations in evaluating post-immunotherapy responses, new biomarkers, including metabolic activity of the gut microbiome, metabolic tumor volume (MTV), and total lesion glycolysis (TLG), show promising potential for assessing therapeutic responses [2]. This narrative review aims to critically appraise the current role of 18F-FDG PET/CT in assessing immunotherapy response in NSCLC, with a focus on imaging response criteria, interpretative pitfalls, and potential avenues for future research.

## 2. Methods

Although this article is a narrative review, we employed a structured literature search strategy inspired by systematic review methodology. A flow diagram was included to enhance clarity and reproducibility. Article selection was guided by the SANRA (Scale for the Assessment of Narrative Review Articles) criteria to uphold methodological quality. A comprehensive search was conducted in July 2025 across PubMed, Web of Science, Scopus, and the Cochrane Library, targeting studies published between 2013 and 2025 to reflect key developments from the past decade.

The search strategy included the following MeSH terms and keywords: PubMed: “18F-FDG PET/CT” (MeSH Terms), “NSCLC” (MeSH Terms), “immunotherapy response” (MeSH Terms), “RECIST 1.1” (MeSH Terms), or “iRECIST” (MeSH Terms). Web of Science, Scopus, and Cochrane Library: Keywords such as “18F-FDG PET/CT evaluation,” “tumor response to immunotherapy,” “pseudoprogression,” “hyperprogression,” and “immune checkpoint inhibitors” were used, focusing on title, abstract, and keyword fields.

The studies included in this review met the following criteria:

1. Published in English;
2. Conducted on human subjects;
3. Evaluated the use of PET/CT for monitoring immunotherapy response in patients with NSCLC
4. Clinical trials, cohort studies, comparative studies, or reviews specifically addressing metabolic imaging and response evaluation frameworks (e.g., RECIST 1.1, iRECIST, PERCIST).

The exclusion criteria included the following:

- Articles which are not published in English;
- Involved animals or preclinical research;
- Did not assess 18F-FDG PET/CT in the context of NSCLC immunotherapy;
- Used alternative diagnostic techniques (e.g., CT, MRI or bronchoscopy) without PET/CT correlation;
- Focused on cancers other than NSCLC or non-oncological conditions;
- Were case reports/commentaries/editorials/opinion pieces without original data included;
- Did not provide measurable outcomes for tumor response assessment
- Reviews and meta-analyses were used only for contextual purposes and to identify relevant primary studies, without quantitative data extraction, to avoid data duplication;
- Conference abstracts were excluded from the analysis to ensure data quality and reproducibility.

**Figure 1:**
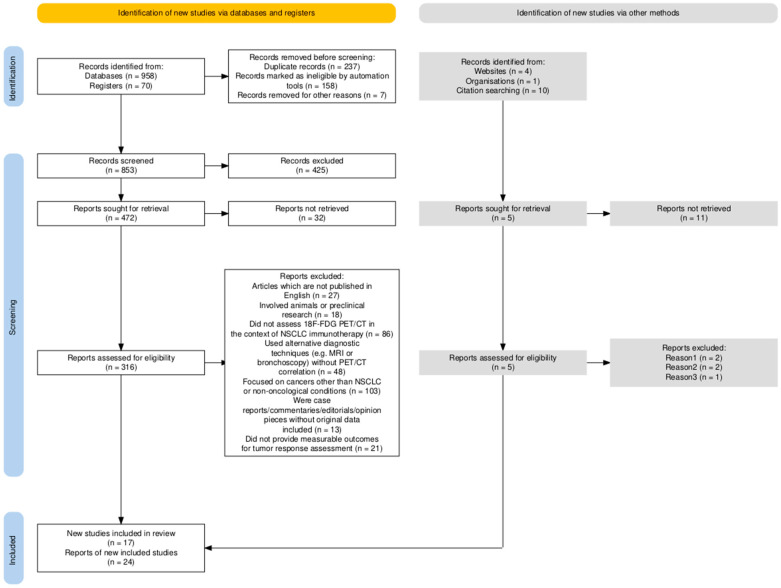
Flow diagram summarizing the structured literature search and selection process used in this narrative review. The figure illustrates the number of records identified, screened, excluded, and included in the final synthesis, adapted according to SANRA guidelines for qualitative reviews. SANRA—Scale for the Assessment of Narrative Review Articles; NSCLC—non-small cell lung cancer; PET/CT—positron emission tomography/computed tomography.

The identification and selection process adhered to SANRA principles, ensuring transparency and reproducibility. The flowchart below illustrates the employed search strategy, implemented filters and article selection process. To complement this, a step-by-step algorithm outlining the review methodology, based on the SANRA guidelines, is presented in Figure 1.

## 3. Tumor Response Criteria: Clinical Relevance and Limitations

### 3.1. RECIST 1.1: Strengths and Weaknesses

The RECIST 1.1 (Response Evaluation Criteria in Solid Tumors) established by the World Health Organization (WHO) and updated in 2009, provides a standardized framework for assessing tumor burden and therapeutic response in oncology, based on anatomical changes in target and non-target lesions [20,21,22,23]. Responses are classified into four categories: complete response (CR), partial response (PR), stable disease (SD) and progressive disease (PD), according to tumor size changes over time compared to baseline measurements. As a reliable, standardized and reproducible method, RECIST 1.1 supports clinicians in objectively assessing treatment efficacy. It also simplifies the comparison of imaging-based evaluations, aiding in clinical decision-making and contributing to more accurate and consistent study outcomes [20,22,23].

While RECIST 1.1 ensures reproducibility and consistency across studies, it was primarily designed for cytotoxic therapies and does not account for atypical response patterns often seen under immunotherapy, such as pseudoprogression or dissociated responses [22,24,25,26]. These phenomena can result in early misclassification of immune-related response as true disease progression, potentially leading to the premature cessation of effective therapies [27,28,29].

Despite its broad applicability, RECIST 1.1 is increasingly insufficient in the immunotherapy era due to its inability to distinguish true progression from immune-modulated responses.

In RECIST 1.1, tumor response is assessed anatomically by tracking predefined target and non-target lesions over time. Selection rules for measurable lesions and inclusion thresholds are summarized in Table 1 [24,25,26].

Based on changes in the sum of diameters (SOD) over time, RECIST 1.1 defines four standardized tumor response categories, as presented in Table 2 [24,25,26].

**Figure 2:**
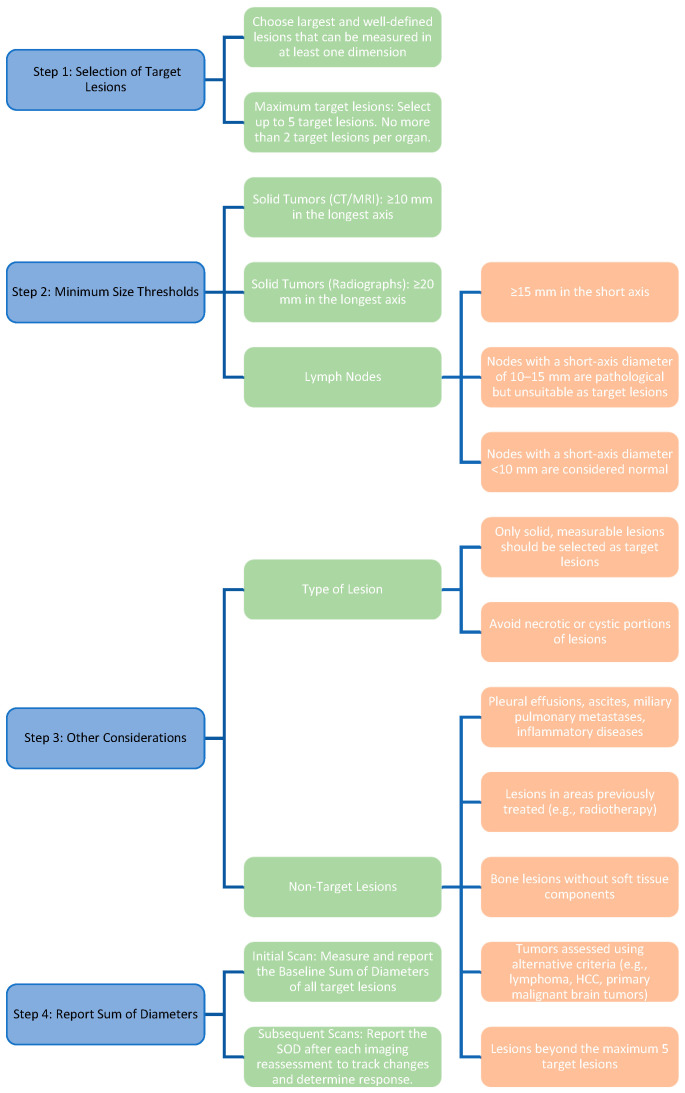
Diagnostic algorithm for the selection and monitoring of target lesions according to RECIST 1.1. The diagram summarizes the sequential steps for identifying measurable target lesions, including selection rules, minimum size thresholds, lesion type considerations, and reporting of the sum of diameters (SOD) for treatment response assessment. Abbreviations: RECIST—Response Evaluation Criteria in Solid Tumors; CT—computed tomography; MRI—magnetic resonance imaging; SOD—sum of diameters.

Figure 2 shows a simplified workflow for selecting and monitoring target lesions under RECIST 1.1.

### 3.2. iRECIST: A Partial Solution to Immunotherapy Challenges

iRECIST (2017) adapts RECIST 1.1 to immunotherapy by introducing immune-specific categories—iUPD and iCPD—to allow for treatment continuation beyond apparent progression and to confirm or refute pseudoprogression on follow-up imaging [22,27,29,30,31]. This prevents premature discontinuation of potentially effective ICIs in clinically stable patients [27,28,29,30].

iRECIST refines recognition of immune-related dynamics, but routine implementation and harmonization with metabolic criteria need further validation [27,28,29,30,31].

## 4. iRECIST: Atypical Response Patterns in Immunotherapy: Clinical and Imaging Insights

While RECIST 1.1 remains the anatomical standard, it was not designed for immune-related patterns. iRECIST extends RECIST 1.1 with immune-specific categories to capture pseudoprogression and delayed responses and to allow for treatment beyond apparent progression in clinically stable patients [22,27,28,29].

The primary goal of the iRECIST criteria is to provide clinicians with a standardized and consistent approach to evaluate tumor responses in patients treated with immunotherapy, particularly ICIs. iRECIST aims to account for the unique immune-related response patterns, enabling a more accurate assessment of treatment outcomes [29].

Thus, new concepts have been introduced. ICIs have been associated with distinct response patterns that deviate from conventional responses. These patterns include pseudoprogression (PPD), dissociated response (DR) and hyperprogression (HPD). Another distinctive feature of ICIs, compared to cytotoxic chemotherapy or molecularly targeted therapies, is their ability to induce long-lasting responses in a subset of patients [23,30].

### 4.1. Pseudoprogressive Disease

This tumor response pattern has led to the development of new response criteria for patients treated with immunotherapy. Pseudoprogressive disease (PPD) typically occurs within the first 4–6 weeks after initiating treatment with ICIs, though it may manifest several months later [23,31,32]. As it is not a diagnosis of proliferative disease, it is critical to continue potentially effective treatment [33]. Due to variations in response criteria and the degree of response required to confirm pseudoprogression (e.g., stable disease, partial response, or complete response) following initial tumor progression, the exact definition of pseudoprogression has not been uniformly established [31,34].

iRECIST builds upon RECIST 1.1 by introducing additional categories and modifications to account for immune therapy-specific reactions. It defines 4 response categories to better capture these unique patterns of tumor response as summarized in Table 3, which provides an overview of their definitions, radiologic criteria, and clinical management considerations [23,29,35].

### 4.2. Hyperprogression

Hyperprogression refers to the paradoxical acceleration of tumor growth kinetics in response to the initiation of immunotherapy with ICIs [36,37]. There is currently no universally accepted consensus or clear definition of hyperprogression. One proposed criterion is an increase of more than twofold in the tumor growth rate (TGR) after starting immune therapy [38,39]. This phenomenon has been observed in approximately 9% of patients following the initiation of immunotherapy, across various cancer types. The biological mechanisms underlying hyperprogression remain unclear. Evidence suggests that several factors may contribute to this phenomenon, including MDM2 gene amplification, prior radiotherapy or ablation, alterations in EGFR pathways, advanced age, and high mutational burden. Local inflammation and dysregulation of other immune checkpoints may also facilitate tumor escape [40]. Interestingly, the incidence of hyperprogression appears to be lower in patients with non-small cell lung cancer (NSCLC) treated with a combination of anti-PD-1 inhibitors and double platinum chemotherapy, compared to those receiving ICIs as monotherapy [1,34,41].

From the perspective of 18F-FDG PET/CT, there is currently no solid evidence to predict hyperprogression. However, an increased risk of hyperprogression has been noted in patients with non-small cell lung cancer (NSCLC) treated with ICIs who exhibit a higher MTV and an elevated neutrophil-to-lymphocyte ratio (NLR). These findings suggest the potential for developing a multiparametric model to optimize the prediction of tumor response [1,42].

### 4.3. Dissociated Response

In morphological imaging, within the context of iRECIST criteria, the dissociated response also referred to as a mixed response, describes a phenomenon observed during immunotherapy. It is characterized by the coexistence of two contradictory types of lesions in the same patient:

○ Complete Response (CR) or Partial Response (PR) in some lesions;
○ Co-occurrence of other lesions showing no response to immunotherapy (Stable Disease [SD] or Progressive Disease [PD]) [1,43,44].

It remains unclear whether the concurrent presence of lesion progression and regression is sufficient to diagnose a dissociated response, or if specific thresholds, or if specific thresholds (e.g., ≥20% PD and ≥30% PR) are required [45,46,47,48,49].

In studies using 18F-FDG PET/CT, the term immune dissociated response (iDR) has been defined as an imaging improvement in some hypermetabolic lesions combined with the worsening of others, in contrast to immune homogeneous PD. For PET/CT, the definition of dissociated response derives from the PET Response Criteria in Solid Tumors (PERCIST). It is characterized by a decrease of more than 30% in metabolism in some tumor lesions, alongside a relative increase of more than 30% in others [45,50]. This seemingly contradictory behavior reflects the unique mechanisms of immunotherapy rather than true disease progression [1,51]. This phenomenon can be explained by multiple theories and pathophysiological factors, such as differences in the TME across distinct metastatic sites and the genomic heterogeneity of tumors [1,43]. Additionally, differences in tissue penetration of anticancer therapies at the lesion level have been proposed as potential contributors to the dissociated response [45,46].

Cancer cells can undergo clonal evolution, progressing from a single progenitor cell to more aggressive and treatment-resistant subclones. This phenotypic and genotypic heterogeneity represents a poor prognostic factor for survival. It may also explain the dissociated response, particularly under targeted therapies that exert selective pressure on specific tumor clones. Furthermore, the heterogeneity of the immune tumor microenvironment may actively influence therapeutic responses, thereby accounting for the differential imaging responses among lesions [43,45].

While some evidence suggests that the DR is associated with treatment efficacy in approximately 10% of patients with advanced lung cancer treated with ICIs, current response criteria often classify this imaging pattern as PD [1,51]. Clinicians should also consider certain oncological traps in the differential diagnosis of dissociated response, such as: synchronous cancers, treatment-related side effects (e.g., sarcoidosis-like reactions), specific response patterns (e.g., progression of a single metastatic lesion), and inflammation-induced contrast agent uptake in imaging assessments with 18F-FDG PET/CT [1,44].

Survival outcomes were found to be similar between patients with atypical responses and those with non-progressive disease, even though DR and pseudoprogression were not separately evaluated. Notably, higher survival rates have been observed in patients with dissociated response compared to those with progressive disease. This finding has been supported by both the studies of Tozuka et al. [46] and Tazdait et al. [52], suggesting that the prognosis for patients with dissociated response is most likely intermediate, positioned between that of patients with non-progressive disease and those with progressive disease [45,46,52].

A study involving 235 patients with advanced lung cancer treated with ICIs compared survival outcomes between patients who continued ICI therapy after the diagnosis of a DR and those who switched to alternative anticancer therapies. DR was observed in 20–34% of patients treated with ICIs. Propensity score matching (PSM) was used to minimize potential confounding factors. Among the results, 52 patients were classified as having a dissociated response. After PSM, the post-DR cohort that continued ICI therapy demonstrated a significantly longer median overall survival compared to the post-DR cohort that discontinued ICIs: 10.63 months (95% CI 6.27–NA) versus 4.33 months (95% CI 1.77–NA), respectively (*p* = 0.016). Within the limitations of this retrospective, single-center analysis, it was demonstrated that clinically stable patients deemed eligible by their clinicians to continue ICI therapy after the diagnosis of DR showed improved overall survival compared to those who opted to discontinue ICIs [47].

In a retrospective study involving 107 patients, the characteristics and outcomes of immunotherapy were examined in patients diagnosed with advanced non-small cell lung cancer (NSCLC) who were treated with nivolumab monotherapy and exhibited a DR as a treatment outcome. The analysis also included patients who had previously received chemotherapy or tyrosine kinase inhibitors (TKIs). Tumor response in each organ was evaluated using RECIST 1.1 criteria during the first imaging assessment. The study further investigated treatment outcomes and compared overall survival (OS). Patients who demonstrated DR on imaging had a significantly longer overall survival compared to those with progressive disease (46.9 months vs. 8.2 months, *p* = 0.038). These findings suggest that some patients may benefit from continuing treatment with nivolumab to achieve greater overall survival. The recognition of dissociated response highlights the potential for achieving optimal outcomes in patients undergoing immunotherapy, which might otherwise be prematurely interrupted due to treatment discontinuation [53].

### 4.4. Durable Response

In the context of iRECIST, a durable response refers to a sustained tumor remission or positive response following immunotherapeutic treatment, such as immune complete response or immune partial response, which persists over an extended period, even after discontinuation of ICIs [2]. This phenomenon is attributed to a tumor-specific immune response, often observed in patients who discontinue ICIs due to immune-mediated adverse events, even when treated with systemic corticosteroids, as the tumor response is frequently maintained [2,27].

This concept is particularly significant in immunotherapy, where treatment effects differ from those of conventional therapies in both mechanism of action and duration. Targeted therapies, aimed at blocking oncogenes, have proven effective in inducing rapid tumor responses, but these responses are typically not durable [1]. In contrast, immunotherapy strategies have the potential to induce delayed but durable responses, explained by the immune infiltration of tumor-specific T cells. Such durable responses are observed in 10–20% of patients [1].

The specific characteristics of a durable response are detailed in Table 4, highlighting key elements such as persistence, long-term efficacy, and clinical impact [53].

A meta-analysis of 19 studies demonstrated the presence of durable responses in 25% of patients treated with ICIs, a rate 2.3 times higher compared to patients not treated with ICIs (11%). Durable responses were more frequently observed in patients treated with anti-PD-1/PD-L1 agents compared to those treated with anti-CTLA4 agents [54]. Consequently, itRECIST recommends a window of 4 to 12 weeks for adequate tumor response assessment, compared to 4 to 8 weeks for immune RECIST (iRECIST) [1].

## 5. Comparison of RECIST 1.1 vs. iRECIST: Differences and Similarities

**Figure 3:**
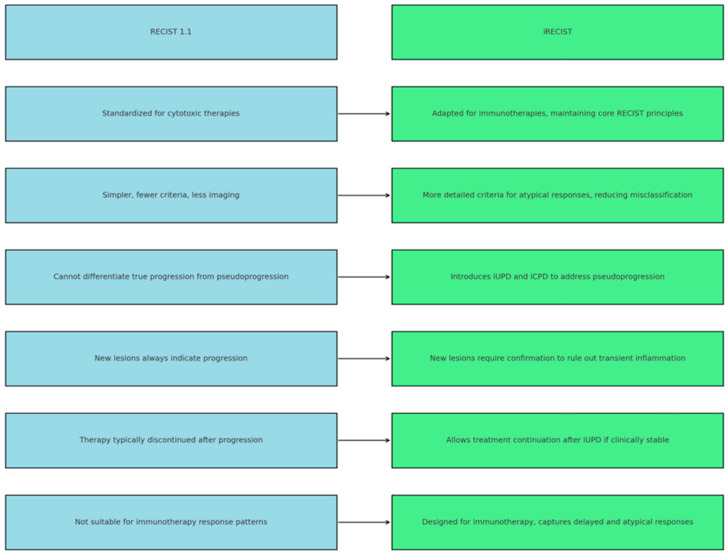
Comparison between RECIST 1.1 and iRECIST in evaluating tumor response to immunotherapy. The diagram highlights key differences between RECIST 1.1 and iRECIST. While RECIST 1.1 was developed for cytotoxic therapies, iRECIST adapts its principles for immunotherapy, introducing immune-specific categories (iUPD, iCPD) to capture pseudoprogression and delayed responses. Abbreviations: RECIST—Response Evaluation Criteria in Solid Tumors; iRECIST—immune-modified RECIST; iUPD—immune unconfirmed progressive disease; iCPD—immune confirmed progressive disease.

Figure 3 illustrates a comparison between RECIST 1.1 and iRECIST, highlighting the differences between the two sets of criteria used for tumor response assessment. RECIST 1.1 is the standard method for evaluating tumor response to chemotherapy, featuring simpler criteria, but with limitations in detecting pseudoprogression and atypical responses to treatment. In contrast, iRECIST is specifically adapted for immunotherapies, introducing the concepts of iUPD and iCPD to better manage pseudoprogression, allowing for treatment continuation under certain conditions, and providing a more accurate assessment of immunotherapy response.

To better highlight the main distinctions between RECIST 1.1 and iRECIST, Table 5 provides a comparative overview summarizing their key features, strengths, and limitations in evaluating tumor response to treatment [22,26,27,55,56,57,58,59].

## 6. What Is the Role of PET/CT in Managing Patients Undergoing Immunotherapy for Lung Cancer?

In patients with non-small cell lung cancer (NSCLC) diagnosed at any stage, PET/CT plays a central role in disease staging and monitoring the treatment response. Additionally, PET/CT is used to evaluate the efficacy of treatment (immunotherapy, chemotherapy, or radiotherapy), to differentiate between active tumor tissue and scar tissue/fibrosis, and to enable the early detection of tumor progression or recurrence. Using PET/CT, the most commonly utilized response criteria are PERCIST 1.0 and EORTC criteria. Both were developed prior to the era of ICIs and are based on models of metabolic changes observed after chemotherapy and/or cytotoxic therapy [2].

Thus, the Positron Emission Tomography Response Criteria in Solid Tumors (PERCIST) are guidelines for the evaluation of solid tumors using positron emission tomography (PET/CT). These guidelines provide functional information [60] by focusing on the assessment of tumor metabolic activity, measured through the uptake of the radiotracer FDG-fluorodeoxyglucose, with the primary parameters including the following:

- SUV (Standardized Uptake Value): A measure of the metabolic intensity of the lesion.
- SUL (Standardized Uptake Value Lean Body Mass): An updated version of SUV, representing a standardized uptake value corrected for lean body mass.
- Metabolic dimension: The volume of the tumor exhibiting high metabolic activity [53,60].

A critical challenge in interpreting and comparing metabolic PET parameters across studies lies in the substantial technical heterogeneity of image acquisition and quantification protocols. Variations in uptake time after tracer injection, scanner calibration, reconstruction algorithms, and SUV normalization methods (e.g., by body weight, lean body mass, or plasma glucose) can markedly influence quantitative parameters such as SUV, MTV, and TLG. Additional differences in reconstruction filters, segmentation thresholds, and quantification software may further affect response classification and prognostic interpretation. Therefore, harmonization of acquisition protocols and adherence to standardized imaging procedures are essential to ensure reproducibility, reliability, and comparability of PET-derived biomarkers [2,16,23].

To ensure methodological consistency and reproducibility across studies, future PET/CT investigations should follow standardized acquisition and quantification protocols. Consensus guidelines such as the EANM/EARL harmonization standards and the PERCIST recommendations emphasize the importance of consistent uptake time (around 60 ± 5 min), scanner cross-calibration, harmonized reconstruction algorithms, and predefined methods for MTV and TLG quantification. Adhering to these minimum standards will improve comparability between studies and support the integration of metabolic biomarkers into clinical decision-making [2,16,23].

The PERCIST classification of tumor response comprises four categories: complete metabolic response (CMR), partial metabolic response (PMR), progressive metabolic disease (PMD), and stable metabolic disease (SMD), each with the following characteristics:

- Complete Metabolic Response (CMR):
  ○ Complete resolution of abnormal metabolic activity detected by the radiotracer 18F-FDG in the target lesion under evaluation. The SUV is comparable to that of normal tissues.
  ○ Disappearance of all other metabolically active lesions.
  ○ No new suspicious 18F-FDG lesions.
- Partial Metabolic Response (PMR):
  ○ A reduction of >30% in the measurable peak metabolic activity of the tumor detected by 18F-FDG, with an absolute decrease in SUL value by at least 0.8 SUL units (indicating a >30% reduction in metabolic activity in the most active lesion SUVmax).
  ○ No increase of >30% in SUL or lesion dimensions in any of the remaining lesions.
  ○ No emergence of new metabolically active lesions.
- Stable Metabolic Disease (SMD):
  ○ Changes below the diagnostic threshold for CMR, PMR, or metabolic progression.
  ○ No appearance of new metabolically active lesions.
- Progressive Metabolic Disease (PMD):
  ○ An increase in the SUL peak of 18F-FDG by >30% or by >0.8 SUL units compared to the baseline imaging, with characteristics consistent with cancer rather than infection or treatment effects.
  ○ A visible increase in the degree of 18F-FDG uptake by the tumor or the appearance of new metabolically active lesions typical of cancer, excluding infection or treatment-related characteristics [24,25,60,61].

**RECIST 1.1 Criteria Adapted for 18F-FDG PET/CT:**

Although RECIST 1.1 primarily relies on anatomical measurements (CT/MRI), PET/CT can be used complementarily to assess metabolic changes and identify new metabolically active lesions. The adapted criteria include the following:

○ Complete response: Disappearance of all lesions and metabolic activity.
○ Partial response: A reduction of >30% in the size of lesions and metabolic activity.
○ Stable disease: No significant reduction or increase in the size or metabolic activity of lesions.
○ Progressive disease: An increase of >20% in the size or metabolic activity of lesions, or the appearance of new metabolically active lesions [24,60,61].

## 7. Are There Alternatives to PERCIST Criteria?

In patients undergoing immunotherapy, interpreting 18F-FDG PET/CT necessitates awareness of both typical and atypical patterns of disease progression. Consequently, several alternative metabolic response criteria have been proposed as substitutes for PERCIST. While PERCIST criteria are typically recommended for the evaluation of molecularly targeted therapies and chemotherapy, their applicability to immunotherapy remains an area of ongoing research. Currently, the evidence base remains insufficient to determine the most appropriate method for response classification in immunotherapy. Moreover, the effect of these response criteria on long-term patient outcomes has not been prospectively confirmed in randomized clinical trials [23]. Clinical series have reported that atypical responses, including pseudoprogression and dissociated response, occur in up to 10% of NSCLC patients under PD-1/PD-L1 blockade, highlighting the importance of confirmatory imaging and multidisciplinary assessment [42,51]. Proposed alternatives to PERCIST are discussed below.

### 7.1. PERCIMPT (PET Response Criteria for Immunotherapy in Malignant Pleural Tumors)

To address the limitations of PET/CT imaging, the PERCIMPT criteria were developed to evaluate tumor response to immunotherapy, particularly in malignant pleural tumors (e.g., mesothelioma) [62,63]. The principles underlying PERCIMPT involve the use of PET/CT to measure radiotracer uptake (SUVmax and SUVmean) in tumors, with metabolic changes serving as the primary marker of response to specific treatment. PERCIMPT is adapted to account for potential pseudoprogressions that are common during immunotherapy [62,63]. A key feature of PERCIMPT is its recognition of atypical responses, such as a temporary increase in metabolic activity or enlargement of lesions before regression. It includes a reassessment period of 4 to 8 weeks to confirm progression [62,63]. PERCIMPT emphasizes that changes in the absolute number of 18F-FDG-avid lesions are more reliable indicators of clinical outcomes during immunotherapy than standardized uptake values (SUV). According to the PERCIMPT criteria, an isolated increase in SUV does not inherently signify disease progression. Likewise, the detection of a single new hypermetabolic lesion on [18F]-FDG PET/CT should not be interpreted as progression, as is commonly performed under conventional PERCIST or EORTC frameworks. Instead, disease progression is more accurately characterized by the emergence of at least four new 18F-FDG-avid lesions. Notably, this threshold may be reduced when the newly detected lesions exhibit a larger functional diameter [23].

### 7.2. imPERCIST (Immune-Modified PERCIST)

The aim of imPERCIST is to adapt the PERCIST (PET Response Criteria in Solid Tumors) framework to address the unique challenges posed by immunotherapy in oncology, such as slow progression or pseudoprogression. Unlike conventional criteria, the appearance of new lesions alone is not sufficient to classify a patient as having progressive disease [23,62,63]. The principles of imPERCIST are based on PERCIST 1.0, but incorporate the distinct features of immunotherapy responses [62,63]. Metabolic response is assessed by summing the peak standardized uptake values, normalized to lean body mass (SULpeak), for up to five target lesions identified on both baseline and follow-up PET/CT scans. No more than two lesions are selected per organ. In follow-up scans, the target lesions are chosen based on the highest metabolic activity, which may differ from those selected at baseline. Progressive metabolic disease (PMD) is defined as an increase of more than 30% in the total SULpeak. However, the mere presence of new lesions does not, on its own, indicate PMD. Such lesions are included in the SULpeak calculation only if their uptake surpasses that of the existing target lesions or if fewer than five target lesions were initially identified [23]. This approach allows for reassessment in cases of suspected initial progression, helping to exclude pseudoprogression. A key feature of imPERCIST is its recognition of atypical responses, such as a temporary increase in metabolic activity or lesion size before regression. It incorporates a reassessment period of 4–8 weeks to confirm progression [62,63].

### 7.3. PERCIST (Immune PET Response Criteria in Solid Tumors)

Recently, some studies have adapted PERCIST to include a clinical “wait-and-see” approach, resulting in iPERCIST, with the primary goal of extending PERCIST criteria to focus on the response of solid tumors to immunotherapy [62,63]. The principles underlying iPERCIST are similar to those of imPERCIST, but provide more specific criteria tailored to immunotherapeutic treatments, including evaluation of SUV in both target and non-target lesions, while recognizing immunologic progression and delayed responses associated with immune mechanisms [62,63]. According to iPERCIST, patients presenting with new lesions or a dimensional increase >30% in SULpeak or in the SULpeak of the most FDG-avid lesions are classified as having unconfirmed progressive disease. A follow-up imaging evaluation after 4–8 weeks is required to confirm progressive metabolic disease [23]. Recent studies have shown that in advanced-stage lung cancer patients with unconfirmed progressive metabolic disease based on initial PET/CT imaging, a follow-up confirmatory scan reclassifies approximately one-third of these patients as exhibiting atypical response patterns (e.g., pseudoprogression or dissociated response). These patients ultimately benefit from continued treatment with ICIs. This highlights the potential pitfalls of prematurely discontinuing immunotherapy based on an initial diagnosis of unconfirmed progressive metabolic disease, a risk particularly pronounced when using PET/CT due to its heightened sensitivity in detecting immune-related cellular activity [23]. The unique features of iPERCIST explicitly integrate immunologic analysis of lesions, adjusting the definitions of metabolic progression to account for inflammatory activity induced by immunotherapy [62,63,64,65]. Recent prospective evaluations demonstrated that PET-based immune criteria such as imPERCIST and iPERCIST outperform RECIST and iRECIST in predicting both overall and progression-free survival in NSCLC under immunotherapy [63,65,66].

To better understand modern methods for evaluating tumor response to current oncologic treatments, particularly immunotherapies, Table S1 provides a comparison of the PERCIMPT, imPERCIST, and iPERCIST criteria. These evaluation systems have been designed to assess treatment efficacy in specific ways, accounting for the unique characteristics of metabolic and, especially, immunologic responses, as well as distinct phenomena such as pseudoprogression and delayed response. Each of these methods introduces improvements and adaptations compared to earlier standards (e.g., RECIST 1.1 or PERCIST criteria), reflecting the complexity of contemporary therapies [62,63,64,65].

### 7.4. Clinical Implications and Guidance for Decision-Making

In clinical practice, PET/CT findings should be interpreted in conjunction with iRECIST to guide management during immunotherapy. A decrease or stabilization in metabolic activity (partial or stable metabolic response per PERCIST, imPERCIST, or iPERCIST) supports the continuation of immune checkpoint inhibitors. In cases of suspected pseudoprogression, where metabolic activity decreases or remains stable despite anatomical enlargement, therapy should be maintained and PET/CT repeated after 4–8 weeks for confirmation. Confirmed metabolic progression (increase in SULpeak, MTV, or TLG ≥30%, or new unequivocal FDG-avid lesions) may indicate the need to modify or discontinue treatment, particularly if associated with clinical deterioration. Conversely, early and marked increases in metabolic burden consistent with hyperprogression should prompt treatment discontinuation. These practical recommendations align with iRECIST and PET-adapted criteria and are supported by current EANM/EARL harmonization guidance [16,22,23,48,64,65,66,67,68,69,70].

### 7.5. Challenges of FDG Uptake Interpretation and Differentiation Strategies

A major limitation of 18F-FDG PET/CT in the context of immunotherapy is that FDG uptake is not tumor-specific. Increased metabolic activity may also reflect inflammatory processes, immune-related adverse events (irAEs), or infection, all of which can mimic disease progression or pseudoprogression. Common irAEs such as pneumonitis, colitis, thyroiditis, and sarcoid-like granulomatous reactions may present with elevated FDG uptake, potentially confounding response assessment. Differentiation between inflammatory and neoplastic uptake relies on careful pattern analysis and clinical correlation. Tumor-related uptake is typically focal, asymmetric, and located in known disease sites, whereas inflammatory or immune-related uptake often appears diffuse, symmetric, and associated with non-target organs (e.g., mediastinal or hilar lymph nodes, bowel, thyroid, or lungs). Temporal assessment with follow-up imaging and integration of clinical and biochemical data can further help clarify equivocal findings. Awareness of these patterns is essential to avoid misclassification of treatment response. Novel immune-PET tracers targeting immune-related molecules such as CD8, PD-1, and PD-L1 are currently under investigation and may offer improved specificity for distinguishing tumor metabolism from immune activation. Although still limited to early-phase studies, these agents represent promising tools for refining immunotherapy response assessment [13,15,23,48,49].

### 7.6. Cost-Effectiveness, Availability, and Potential Risks of PET-Based Monitoring

Although PET/CT offers valuable insights for immunotherapy assessment, its broader implementation must consider cost and resource limitations. The high cost and variable availability of PET/CT scanners may restrict routine use, especially in low-resource settings. Repeated imaging also increases cumulative radiation exposure and the risk of false-positive findings due to immune-related inflammation. Therefore, PET-based monitoring should be applied selectively, supported by standardized protocols and cost-effectiveness evidence to ensure sustainable clinical benefit [2,16,23,49].

### 7.7. Radiomics and Artificial Intelligence in Immunotherapy Response Assessment

Recent advances in radiomics and artificial intelligence (AI) have opened new perspectives for quantifying tumor heterogeneity and predicting immunotherapy outcomes beyond conventional PET metrics such as SUV, MTV, and TLG. Radiomics allows for the extraction of high-dimensional quantitative features from PET/CT images, reflecting underlying biological processes such as metabolism, necrosis, and immune cell infiltration. Machine learning and deep learning models can integrate these features with clinical and genomic data to identify complex imaging signatures associated with response or resistance to immune checkpoint inhibitors. Preliminary studies suggest that radiomics-based biomarkers may outperform traditional parameters in predicting progression-free and overall survival in NSCLC patients treated with ICIs. However, these approaches remain limited by the lack of standardized feature extraction protocols, small sample sizes, and insufficient external validation. Future research integrating PET-based radiomics with AI-driven analytics could enable personalized prediction of immunotherapy efficacy and contribute to the development of automated response assessment tools [71,72,73].

## 8. Comparison of Tumor Response Evaluation Criteria in Major Studies Conducted over the Last Five Years

In the past five years, several medical studies have been conducted comparing tumor response evaluation criteria for treatment with ICIs in patients with non-small cell lung cancer (NSCLC). Table 6 is a comparative summary of the results from five studies conducted in the last five years, encompassing a total of 438 NSCLC patients treated with ICIs. The studies evaluate tumor responses to immunotherapy using various criteria, including RECIST 1.1, iRECIST, PERCIST 1.0, imPERCIST, and iPERCIST. The primary objective of these studies is to identify the most effective methods for evaluating tumor response, particularly in the context of immunotherapy, which can produce atypical responses such as pseudoprogression or delayed response [66,67,68,69,70]. A comprehensive summary of the main response assessment frameworks (RECIST 1.1, iRECIST, PERCIST, imPERCIST, and iPERCIST), including their methodological thresholds, timing rules, strengths, limitations, and diagnostic performance metrics, is provided in Supplementary Table S2 [16,22,27,48,63,64,65,70].

Despite its value, iRECIST increases operational complexity, extends evaluation timelines, and lacks broad prospective validation; integration with PET-based metabolic criteria also remains under study. Recent NSCLC series show that iRECIST better captures atypical immune responses and correlates more closely with PFS than RECIST 1.1 [66,70].

Table 6 serves as a tool for the easier selection of tumor response evaluation criteria for immunotherapy in NSCLC. By comparing the benefits and limitations of each criterion across the five selected studies conducted over the past five years, encompassing a total of 438 patients, it highlights the central role of metabolic and immunologic criteria in optimizing the management of patients treated with immunotherapy. Several observations provide a detailed perspective on the advantages and disadvantages of each criterion, aiding in the interpretation of the data in the context of treating patients with NSCLC [66,67,68,69,70].

A. **Relevance of the criteria for immunotherapy:** Classical criteria, such as RECIST 1.1, implemented in clinical practice in 2009, were designed for traditional cytotoxic and targeted therapies and are not ideal for immunotherapy due to atypical immune responses (e.g., pseudoprogression or delayed response). As an update, iRECIST was introduced to allow for follow-up monitoring of suspected progression to identify diverse immune responses. Metabolic imaging criteria, such as PERCIST and iPERCIST, provide additional information and a different perspective by analyzing tumor metabolic changes, which are often correlated with immunotherapy [66,68,69].
B. **Pseudoprogression** refers to transient immune responses where immunotherapy-induced tumor infiltration by immune cells (e.g., T lymphocytes) and the presence of local inflammatory cells initially result in apparent tumor progression, followed by dimensional reduction. These phenomena occur more frequently in patients treated with PD-1/PD-L1 inhibitors. Studies suggest pseudoprogression rates range between 4% and 7%. The application of updated criteria facilitates the early identification of these phenomena, preventing the premature discontinuation of potentially effective treatments [66,67,69].
C. Concordance Between Criteria: The studies report moderate concordance between anatomical criteria (RECIST 1.1 and iRECIST) and metabolic criteria (PERCIST and iPERCIST), particularly in early responses. Predictors of treatment response include the following:
  - Tumor size, which serves as the primary predictor in anatomical criteria (RECIST 1.1 and iRECIST) [68,70].
  - Maximum Standardized Uptake Value (SULmax), used in metabolic criteria (PERCIST and iPERCIST) to determine tumor metabolic activity [67,69].
  - Immune response patterns, best assessed with iRECIST and imPERCIST, allowing for a better understanding of tumor dynamics under immunotherapy [66,68].
D. Correlation with Overall Survival (OS) and Progression-Free Survival (PFS): Across the five analyzed studies, metabolic criteria, particularly PERCIST and iPERCIST, demonstrate a stronger correlation with OS and PFS compared to anatomical criteria [66,67,68,69,70].
E. Clinical decisions influenced by the choice of imaging evaluation criteria play a crucial role in determining whether to continue or discontinue immunotherapy. For instance, the use of anatomical criteria may lead to an underestimation of immune tumor responses, potentially resulting in the premature discontinuation of treatment. In contrast, metabolic criteria enable better management of complex cases, particularly those involving atypical responses such as pseudoprogression or delayed response [66,67,68,69,70]. Recent studies have shown that immune-related inflammation may mimic tumor progression on ^18F-FDG PET/CT, emphasizing the potential value of immune-specific tracers such as PD-L1 or CD8-targeted PET agents [23,74,75,76].

## 9. Conclusions

Evaluating tumor response to immunotherapy in non-small cell lung cancer (NSCLC) remains a clinical and imaging challenge, particularly due to atypical tumor response patterns, such as pseudoprogression, hyperprogression and dissociated response. Supporting this effort, 18F-FDG PET/CT offers valuable functional and metabolic insights that complement anatomical imaging, optimize therapeutic management while also helping to avoid the premature discontinuation of effective treatments. Compared to conventional RECIST 1.1, updated criteria such as iRECIST, PERCIST, imPERCIST, and iPERCIST provide a more refined framework for interpreting tumor behavior under immunotherapy. Additionally, the incorporation of PET-derived biomarkers such as MTV and TLG shows promise in enhancing response prediction.

However, the clinical integration of these advanced tools remains limited by the lack of standardized protocols and prospective validation in large trials. Further research is needed to harmonize metabolic response criteria, validate them across diverse patient populations, and assess their predictive value in guiding long-term outcomes. Until then, PET/CT should be considered a complementary modality rather than a definitive standard in immunotherapy response assessment.

## Figures and Tables

**Table 1:** RECIST 1.1 Criteria for Selection and Classification of Target and Non-Target Lesions in Solid Tumors.

Category	Criteria/Description
Number of target lesions	- Select preferably the largest and most well-defined lesions - Lesions must be accurately measurable in at least one dimension - Maximum: **5 target lesions in total**, with **no more than 2 per organ**
Minimum size thresholds	- **Solid tumors (CT or MRI):** ≥10 mm in the longest diameter - **Solid tumors (X-ray):** ≥20 mm in the longest diameter - **Lymph nodes:** ≥15 mm in the short axis 10–14 mm: pathological, but not target-eligible <10 mm: considered normal
Type of lesion	- Only **solid, measurable** lesions should be selected - **Avoid necrotic or cystic components** of lesions when measuring - Lesions should be reproducibly measurable on follow-up imaging
Non-target lesions	Lesions that do **not meet criteria** for target selection, including: - Pleural effusions, ascites, miliary pulmonary metastases - Inflammatory diseases, lymphangitic spread (lung or skin)

Legend. Table summarizing the RECIST 1.1 definitions and inclusion rules for measurable (target) and non-measurable (non-target) lesions. The table specifies size thresholds, lesion type requirements, and reproducibility criteria for response assessment. Abbreviations: CT—computed tomography; MRI—magnetic resonance imaging; RECIST—Response Evaluation Criteria in Solid Tumors.

**Table 2:** Definitions of Tumor Response Categories According to RECIST 1.1 Criteria.

Response Category	Criteria
Complete Response (CR)	All of the following conditions must be met: - Disappearance of all target lesions and non-target lesions. - Reduction in the size of pathological lymph nodes to a short-axis diameter of <10 mm. - No appearance of new lesions.
Partial Response (PR)	All of the following conditions must be met: - A reduction of at least 30% in the SOD compared to the BSD. - Non-progressive disease in non-target lesions. - No appearance of new lesions.
Progressive Disease (PD)	Progression is indicated by any of the following criteria: - Appearance of any new lesion. - A significant increase of at least 20% in the SOD of target lesions, with an absolute increase of at least 5 mm, compared to the lowest recorded SOD (nadir).
Stable Disease (SD)	Criteria for PD or PR are not met

Legend. Table summarizing the four standard tumor response categories defined by RECIST 1.1, based on the measurement of target and non-target lesions. Each response category (CR, PR, SD, PD) is determined by quantitative changes in the sum of diameters (SOD) compared with baseline or nadir values. Abbreviations: CR—complete response; PR—partial response; SD—stable disease; PD—progressive disease; SOD—sum of diameters; BSD—baseline sum of diameters.

**Table 3:** Immune-Related Response Categories According to iRECIST Criteria and Recommended Clinical Actions.

Response Category	Acronym	Definition	Imaging Criteria	Recommended Action	Additional Notes
Immune Unconfirmed Progressive Disease	iUPD	Apparent disease progression compared to the nadir, but not meeting RECIST 1.1 criteria for PD	- Increase in target lesions not meeting PD threshold - Possible new lesions (not definitive) - Requires imaging confirmation	Continue treatment if clinically appropriate; repeat imaging within 4–8 weeks	Allows for the detection of pseudoprogression
Immune Confirmed Progressive Disease	iCPD	Apparent disease progression compared to the nadir, but not meeting RECIST 1.1 criteria for PD	- Further increase in target lesions (≥5 mm) - Progression of non-target lesions - Appearance or growth of new measurable/non-measurable lesions (sum ≥ 5 mm)	Discontinue treatment if clinically indicated	Confirms that initial iUPD was true progression
Immune Complete Response	iCR	Disappearance of all target, non-target, and new lesions	- All lesions resolved - All lymph nodes reduced to ≤10 mm short-axis diameter (same as RECIST 1.1)	Continue treatment or consider discontinuation based on protocol	Equivalent to CR per RECIST 1.1
Immune Partial Response	iPR	Reduction of ≥30% in total tumor burden of target lesions	- Decrease in sum of diameters of target lesions ≥30% - Persistence of non-target lesions or resolution of some target lesions	Continue treatment	Same as PR per RECIST 1.1
Immune Partial Response	iSD	Does not meet criteria for iCR, iPR, or iCPD	- No sufficient shrinkage for iPR/iCR - No sufficient increase for iUPD/iCPD	Continue treatment and monitor	Same as SD per RECIST 1.1

Legend. Table summarizing the immune-related response categories defined by iRECIST for evaluating tumor response to immunotherapy. Each category (iCR, iPR, iSD, iUPD, iCPD) builds upon the standard RECIST 1.1 framework, but incorporates immune-specific patterns such as pseudoprogression and delayed response. Follow-up confirmation of progression (from iUPD to iCPD) is required within 4–8 weeks to avoid premature discontinuation of effective treatment. Abbreviations: iCR—immune complete response; iPR—immune partial response; iSD—immune stable disease; iUPD—immune unconfirmed progressive disease; iCPD—immune confirmed progressive disease; CR—complete response; PR—partial response; SD—stable disease; PD—progressive disease.

**Table 4:** Key characteristics and clinical relevance of durable response to immunotherapy.

Characteristic	Description	Clinical Relevance
Persistence over time	The tumor response (e.g., complete remission or significant tumor reduction) remains stable over a prolonged period, confirmed through serial imaging	Indicates sustained antitumor activity and the potential for long-term disease control
Long-term efficacy	After discontinuation of immunotherapy, the immune system continues to exert antitumor effects, maintaining the response	Suggests durable immunologic memory and prolonged therapeutic benefit without continuous treatment
Lack of progression	No emergence of new lesions or growth of existing ones during the entire follow-up period	Demonstrates ongoing disease stability and supports continued benefit from the initial response
Clinical impact	Patients experience prolonged clinical benefit, including improved survival outcomes and quality of life	Highlights the broader value of durable responses beyond imaging metrics alone
Pseudoprogression followed by response	An initial increase in tumor size due to immune cell infiltration is followed by true tumor shrinkage, with response sustained over time	Requires careful monitoring and interpretation to avoid premature discontinuation of effective therapy

Legend. Table summarizing the defining characteristics of a durable response in patients undergoing immunotherapy. A durable response refers to a long-lasting clinical and radiological benefit that persists beyond treatment discontinuation, often reflecting sustained immune activation and memory. Recognizing these patterns supports better prediction of long-term outcomes and guides treatment continuation or cessation decisions. Abbreviations: none.

**Table 5:** Comparative overview of RECIST 1.1 and iRECIST: key features, strengths, and limitations in tumor response evaluation.

Aspect	RECIST 1.1	iRECIST	Strengths/Limitations Summary
Origin	Developed in 2000 (updated in 2009—RECIST 1.1) to standardize tumor response evaluation for solid tumors treated with cytotoxic therapies	Developed in 2017 as an improvement to RECIST 1.1, addressing atypical response patterns such as pseudoprogression and delayed responses	iRECIST builds upon RECIST 1.1, adapting it for immunotherapy-specific phenomena
Main Objective	Evaluates changes in tumor burden to determine tumor response or progression	Evaluates immune response patterns, including pseudoprogression	iRECIST expands applicability beyond cytotoxic therapies
Lesion Categories	Target lesions Non-target lesions New lesions	Target lesions Non-target lesions New lesions	Structural consistency ensures comparability
Definition of Target Lesions	Measurable lesions selected at baseline to monitor tumor response Up to 5 lesions in total (maximum of 2 per organ) measured along the longest axis	Same as RECIST 1.1 (up to 5 lesions, maximum of 2 per organ)	Maintains methodological uniformity
Definition of Non-Target Lesions	Non-measurable lesions monitored for significant progression or complete response (e.g., ascites, lymphatic spread)	Same definition, but progression requires confirmation with additional imaging	Enhances reliability for immune-related cases
Impact of new lesions	Impact of New Lesions	New lesions are recorded, but do not immediately confirm progression; further confirmation is required to rule out pseudoprogression	Reduces false positives due to inflammatory activity
Complete Response (CR)	Disappearance of all lesions	Same as RECIST 1.1	-
Partial Response (PR)	Reduction >30% in the sum of the diameters of target lesions	Same as RECIST 1.1	-
Stable Disease (SD)	Criteria not meeting CR or PR	Same as RECIST 1.1	-
Progressive Disease (PD)	Increase >20% in the sum of diameters of target lesions or clear progression of target lesions/new lesions	Initial progression is labeled as iUPD and requires imaging confirmation for iCPD	Prevents premature discontinuation of effective immunotherapy
Handling of Pseudoprogression	Not addressed. Progressive disease is diagnosed immediately	Recognizes pseudoprogression: iUPD requiring follow-up imaging for confirmation Reflects lesion growth or new lesions, followed by response or stabilization	Improves diagnostic accuracy under immunotherapy
Additional Immune Categories	Not applicable	Adds immune categories: iCR—immune complete response iPR—immune partial response iSD—immune stable disease iUPD—immune unconfirmed progressive disease iCPD—immune confirmed progressive disease	Allows for refined classification of immune responses
Confirmation of PD	Once progression is identified, no confirmation is required Progression typically leads to treatment discontinuation	Initial progression (iUPD) requires confirmation (iCPD) with follow-up imaging within 4–12 weeks Allows for the continuation of treatment after iUPD if the patient’s clinical condition is stable	Enables continuation of therapy in clinically stable patients
Commonly Applied Therapies	Chemotherapy Radiotherapy Targeted therapies	Immunotherapy Immune checkpoint inhibitors Combination of immune agents	Each criterion suits specific treatment classes
Clinical Decisions	Therapy is often discontinued after confirmation of progressive disease	Therapy may continue after iUPD if the patient’s clinical condition permits, as pseudoprogression or delayed response is possible	Improves management of atypical immune responses
Use in Immunotherapy	Not suitable for immune checkpoint inhibitor treatments	Specifically designed for immune therapies to characterize delayed responses and atypical patterns	iRECIST provides superior clinical applicability
Advantages	Standardized, simple, and reproducible; broadly applicable in conventional oncology	Addresses atypical immune responses; reduces risk of misclassification	Both contribute to consistent evaluation; iRECIST offers higher sensitivity to immune phenomena
Limitations	Cannot distinguish true progression from pseudoprogression; may lead to premature treatment discontinuation	More complex and resource-demanding; confirmation delays may prolong evaluation timelines	Trade-off between simplicity and immunologic precision

Legend. Comparative table summarizing the main conceptual, methodological, and clinical differences between RECIST 1.1 and iRECIST. The table highlights their origins, objectives, lesion classification rules, handling of new lesions, and approaches to pseudoprogression. iRECIST introduces immune-specific categories (iUPD, iCPD) and confirmation timing (4–12 weeks) to better capture atypical immune-related responses. Abbreviations: RECIST—Response Evaluation Criteria in Solid Tumors; iRECIST—immune-modified RECIST; iUPD—immune unconfirmed progressive disease; iCPD—immune confirmed progressive disease; CR—complete response; PR—partial response; SD—stable disease; PD—progressive disease.

**Table 6:** Comparative summary of major studies evaluating tumor response criteria in NSCLC patients treated using immune checkpoint inhibitors.

Authors (Publication Year)	Study Location	Number of Patients	Total Duration	ICI Treatment	Criteria Compared	Key Findings	Reported Pseudoprogressions	Response Predictors	Overall Survival (OS)	Progression-Free Survival (PFS)	Imaging Modality Used	Criteria Concordance	Response Type Analyzed
Nelles et al., 2024 [66]	Germany	252	4 years	Immune checkpoint inhibitors	RECIST 1.1 iRECIST	iRECIST better captured atypical responses. Time to progression (TTP) was significantly longer with iRECIST	Not reported	Immune response patterns	618.3 ± 626.9 days	538.1 ± 617.9 days	CT	High	Atypical immune response
Ayati et al., 2021 [63]	Iran	72	30 months	PD-1 inhibitors	RECIST 1.1 iRECIST PERCIST1.0	All criteria correlated with OS. iRECIST was superior for atypical immune responses	7%	SUL (Standardized Uptake Value)	Significant correlation with OS	Significantly improved	PET/CT	Moderate	Metabolic response
Gupta et al., 2022 [65,68]	India	20	24 months	Nivolumab	iRECIST imPERCIST	High concordance between criteria. iRECIST and imPERCIST better predicted PFS than RECIST 1.1.	Not reported	Tumor size	Not reported	Significantly higher in imPERCIST	PET/CT	High	Complete/partial response
Castello et al., 2019 [69]	Italy	52	36 months	PD-1/PD-L1 inhibitors	RECIST 1.1 iRECIST iPERCIST	iPERCIST provided better predictions for OS and PFS than anatomical criteria. Moderate concordance.	Not reported	Tumor metabolism	Longer OS for iPERCIST responders	Significantly improved	PET/CT	Moderate	Complete/partial response
Beer et al., 2019 [70]	Austria	42	24 months	PD-1/PD-L1 inhibitors	RECIST 1.1 iRECIST PERCIST	Moderate concordance between methods. All predicted OS with no significant differences between them.	Not reported	Tumor size and metabolism	Moderate correlation with OS	Moderate correlation	PET/CT	Moderate	Metabolic response

Legend. Overview of clinical studies conducted between 2019 and 2024 comparing anatomical (RECIST 1.1, iRECIST) and metabolic (PERCIST, imPERCIST, iPERCIST) tumor response criteria in NSCLC patients undergoing immune checkpoint inhibitor therapy. The table summarizes study design, number of patients, treatment type, criteria compared, main outcomes, pseudoprogression rates, predictive biomarkers, and survival endpoints (OS, PFS). Abbreviations: NSCLC—non-small cell lung cancer; ICI—immune checkpoint inhibitor; RECIST—Response Evaluation Criteria in Solid Tumors; iRECIST—immune-modified RECIST; PERCIST—PET Response Criteria in Solid Tumors; imPERCIST—immune-modified PERCIST; iPERCIST—immune PET Response Criteria in Solid Tumors; SUL—standardized uptake value normalized to lean body mass; OS—overall survival; PFS—progression-free survival; PET/CT—positron emission tomography/computed tomography.

## Data Availability

The datasets generated during and/or analysed during the current study are available from the corresponding author on reasonable request.

## Supplementary Materials

The following supporting information can be downloaded at: https://www.mdpi.com/article/10.3390/diagnostics15212754/s1, Table S1: Comparison of PERCIMPT, imPERCIST, and iPERCIST: Advanced Methods for Evaluating Tumor Response to Immunotherapy; Table S2: Comparative Overview of Main Response Assessment Frameworks (RECIST, iRECIST, PERCIST, imPERCIST, iPERCIST) in NSCLC under Immunotherapy.
