# Supplementary material for: The Role of 18F-FDG PET/CT in Monitoring Immunotherapy Response in Non-Small Cell Lung Cancer: Current Evidence and Challenges: A Narrative Review
Source: Diagnostics (Basel). 2025 Oct 30;15(21):2754. doi: 10.3390/diagnostics15212754 (PMC12607320; doi:10.3390/diagnostics15212754)
Supplement: Supplementary file 1 [file diagnostics-15-02754-s001.zip › diagnostics-3928680-supplementary.pdf]

Table S1. Comparison of PERCIMPT, imPERCIST, and iPERCIST: Advanced Methods for Evaluating Tumor Response to Immunotherapy.

Characteristic	PERCIMPT	ImPERCIST	iPERCIST
Definition	A well-defined protocol for evaluating tumor response using PET/CT, specific to cases treated with immunotherapy	Response evaluation criteria in immunotherapy adapted from RECIST 1.1 and PERCIST	A specific version of PERCIST for immunologic treatments, emphasizing inflammation and pseudoprogression
Primary Goal	To assess tumor response to immunotherapeutic treatments, considering PET/CT and tumor metabolic markers	Monitoring the efficacy of immunotherapy by measuring immune phenomena and tumor changes	Providing a robust method to differentiate true progression from pseudoprogression during immunotherapy
Specific Modifications from PERCIST	Considers inflammation associated with immunotherapy	Includes additional criteria for immune effects and pseudoprogression	Adapts metabolic response evaluation to highlight the specific characteristics of immunotherapy
Type of Response Evaluated	Tumor metabolic response and immunologic response.	PET/CT and standard imaging.	PET/CT combined with immunologic analysis.
Primary Tool	PET/CT (fluorodeoxyglucose - FDG).	PET/CT and standard imaging.	PET/CT integrated with immunologic analysis.
Primary Methodology	SUVmax, SUVmean	SUVpeak	Modified SUVpeak for immune-specific changes, with re-evaluation at 4–8 weeks.
Response Categories	● Complete metabolic response (CMR)<br>● Partial metabolic response (PMR)<br>● Stable metabolic disease (SMD)<br>● Progressive metabolic disease (PMD)	● Complete response (iCR)<br>● Partial response (iPR)<br>● Stable disease (iSD)<br>● Progressive disease (iPD)	Similar to imPERCIST but with greater focus on pseudoprogression
Clinical Use	Evaluating immunologic therapies in oncology	Assessing immune response to new therapies	Determining tumor response within the specific context of immunotherapy
Sensitivity to Pseudoprogression	Medium	High	Very high

Unique Features	Focuses entirely on using PET/CT to determine metabolic changes in tumors	Incorporates more immunologic characteristics to estimate complex responses	Optimized for identifying immunotherapy-specific phenomena, such as pseudoprogression and delayed response

Legend. Comparative summary of three PET-based immune-modified response frameworks (PERCIMPT, imPERCIST, iPER-CIST) designed for assessing immunotherapy outcomes. The table emphasizes methodological distinctions, timing rules (4–8 weeks for confirmation), and their relative ability to detect pseudoprogression or delayed immune responses.

Abbreviations: PET/CT—positron emission tomography/computed tomography; FDG—fluorodeoxyglucose; SUV—standardized uptake value; SUL—SUV normalized to lean body mass; CMR—complete metabolic response; PMR—partial metabolic response; SMD—stable metabolic disease; PMD—progressive metabolic disease; iCR—immune complete response; iPR—immune partial response; iSD—immune stable disease; iPD—immune progressive disease.

Table S2. Comparative Overview of Main Response Assessment Frameworks (RECIST, iRECIST, PERCIST, imPERCIST, iPERCIST) in NSCLC under Immunotherapy

Framework	Imaging Modality / Parameter	Key Metric / Thresholds	Timing / Assessment Rules	Strengths	Limitations	Diagnostic Performance (Representative Studies)	Clinical Implications
RECIST 1.1 (Seymour et al., 2017)	CT(anatomic) – sum of longest diameters (SLD)	PR: ≥30% ↓; PD: ≥20% ↑ (≥5 mm absolute)	Baseline + every 6–8 weeks	Standardized, easy to apply	Fails to distinguish pseudoprogression; ignores metabolic changes	Sensitivity ~70%, Specificity ~65% for progression vs PET	Widely used; limited in immunotherapy due to atypical patterns
iRECIST (Seymour et al., 2017)	CT – same metrics as RECIST, adds "unconfirmed	iUPD → requires confirmatory scan ≥4 weeks later	Recognizes pseudoprogression; maintains comparability with RECIST	Relies solely on size; delayed confirmation may miss	Sensitivity 75%, Specificity 68% (Mulkey et al., 2020)	Recommended for trials with immunotherapy; allows continued treatment	

	progression (iUPD)"			hyperprogression		beyond iUPD	
PERCIST 1.0 (Wahl et al., 2009)	PET/CT – SULpeak (lean body mass–normalized)	PMR: ≥30% ↓; PMD: ≥30% ↑ (new lesions = PD)	Baseline vs follow-up (≈4–8 weeks)	Quantitative; reproducible; early metabolic predictor	Sensitive to technical variability (uptake time, reconstruction)	Sensitivity 82–88%, Specificity 75–80% for response prediction (Evangelista et al., 2020)	Enables early response assessment; prognostic for survival
imPERCIST (Ito et al., 2019)	PET/CT – SULpeak; excludes new small inflammatory lesions	PMR: ≥30% ↓ without new lesions suggestive of inflammation	Similar to PERCIST, adapted for immunotherapy	Accounts for immune-related inflammation; improves specificity	Requires expert interpretation; not fully standardized	Specificity up to 90% for pseudoprogression exclusion (Lopci et al., 2021)	Reduces false PD; better correlation with clinical outcomes
iPERCIST / iPERCIST variants (Goldfarb et al., 2019)	PET/CT – MTV, TLG, SULpeak; integrates metabolic and immune response	iUPMD requires confirmation after 4–8 weeks	Combines metabolic and immune-related evaluation	Still evolving; limited prospective validation	Sensitivity 85%, Specificity 82% for outcome prediction (Ayati et al., 2021; Gupta et al., 2022)	Promising tool for distinguishing true progression vs pseudoprogression; suitable for immunotherapy response monitoring	

Legend. Comparison of anatomic (RECIST-based) and metabolic (PERCIST-based) tumor response frameworks applied in NSCLC immunotherapy. The table summarizes key methodological features, quantitative thresholds, timing rules, and diagnostic relevance for evaluating atypical immune-related responses such as pseudoprogression and delayed response.

Abbreviations: RECIST—Response Evaluation Criteria in Solid Tumors; iRECIST—Immune Response Evaluation Criteria in Solid Tumors; PERCIST—PET Response Criteria in Solid Tumors; imPERCIST—Immune-modified PET Response Criteria in Solid Tumors; iPERCIST—Immune PET Response Criteria in Solid Tumors; CT—computed tomography; PET/CT—positron emission tomography/computed tomography; SULpeak—standardized uptake value normalized to lean body mass; SLD—sum of longest

diameters; PMR—partial metabolic response; PMD—progressive metabolic disease; PD—progressive disease; PR—partial response; MTV—metabolic tumor volume; TLG—total lesion glycolysis; ICI—immune checkpoint inhibitor; NSCLC—non-small cell lung cancer.
